# Risk of major systemic complications in patients with cutaneous vasculitis: A retrospective cohort study

**DOI:** 10.1016/j.jdin.2025.04.015

**Published:** 2025-06-11

**Authors:** Arjun Mahajan, John Barbieri, Evan W. Piette

**Affiliations:** a Department of Dermatology, Brigham and Women’s Hospital, Boston, Massachusetts; b Harvard Medical School, Boston, Massachusetts

**Keywords:** artificial intelligence, cutaneous vasculitis, health informatics, natural language processing, risk analysis, skin-limited vasculitis, systemic complications, outcomes

*To the Editor:* In contrast to vasculitides like granulomatosis with polyangiitis and immunoglobulin A vasculitis, which have been associated with renal, vascular, and pulmonary complications,^1^ little is known about the long-term outcomes of patients with skin-limited vasculitis (SLV) and whether there may be associated systemic complications.^1^^,^^2^

Utilizing TriNetX, a Health Insurance Portability and Accountability Act-compliant federated network, we conducted a retrospective cohort analysis with deidentified electronic health records from 128 health care organizations. The study included patient demographic information and diagnoses from October 2, 2014, to October 2, 2024, following strengthening the reporting of observational studies in epidemiology (STROBE) guidelines for cohort studies.

We identified a cohort of 34,905 patients with SLV using a validated International Statistical Classification of Diseases and Related Health Problems, Tenth Revision (ICD-10)-based algorithm, excluding those with any systemic vasculitis diagnosis (Supplementary Supplement 1, available via Mendeley at https://data.mendeley.com/datasets/vc9sbk9cx5/1). A control cohort included 2,119,355 patients with seborrheic keratosis ICD-10 codes. Propensity score matching (1:1, using nearest neighbor greedy matching) balanced cohorts on age, race and ethnicity, sex, and comorbidities, such as hypertension, hyperlipidemia, diabetes, prior malignancy, and smoking status (Table I).

**Table I:** Baseline patient characteristics in skin-limited vasculitis and seborrheic keratosis control cohorts

Characteristic	Before PSM, *n* (%)			After PSM, *n* (%)		
	Skin-limited vasculitis (*n* = 34,905)	Control (*n* = 2,119,355)	SMD	Skin-limited vasculitis (*n* = 33,824)	Control (*n* = 33,824)	SMD
Age at index, mean (SD)	51.6 (21.7)	62.4 (13.7)	0.593	51.9 (21.5)	52.8 (20.4)	0.047
Sex						
Female	20,403 (59.0)	1,130,153 (55.1)	0.079	20,240 (59.0)	20,310 (59.2)	0.004
Male	13,091 (37.9)	825,453 (40.3)	0.049	13,027 (37.9)	12,947 (37.7)	0.005
Unknown gender	1066 (3.1)	94,887 (4.6)	0.080	1063 (3.1)	1073 (3.1)	0.002
Race						
Asian	3134 (9.1)	35,918 (1.8)	0.328	3002 (8.7)	3200 (9.3)	0.020
Black or African American	2277 (6.6)	62,432 (3.0)	0.166	2249 (6.6)	2347 (6.8)	0.011
White	20,415 (59.1)	1,598,875 (78.0)	0.416	20,406 (59.4)	20,414 (59.5)	<0.001
Native Hawaiian or Other Pacific Islander	283 (0.8)	25,159 (1.2)	0.041	283 (0.8)	281 (0.8)	0.001
American Indian or Alaska Native	100 (0.3)	3710 (0.2)	0.022	99 (0.3)	100 (0.3)	0.001
Other race	1121 (3.2)	48,294 (2.4)	0.054	1114 (3.2)	1063 (3.1)	0.008
Unknown race	7230 (20.9)	276,105 (13.5)	0.199	7177 (20.9)	6925 (20.2)	0.018
Comorbidities						
Chronic kidney disease	3869 (11.2)	122,705 (6.0)	0.187	3818 (11.1)	4327 (12.6)	0.046
Type 2 diabetes	5833 (16.9)	250,709 (12.2)	0.132	5811 (16.9)	6411 (18.7)	0.046
Hypertension	12,248 (35.4)	751,129 (36.6)	0.025	12,183 (35.5)	13,041 (38.0)	0.052
Nicotine dependence	3151 (9.1)	166,666 (8.1)	0.035	3139 (9.1)	3425 (10.0)	0.028
Hyperlipidemia	10,164 (29.4)	831,906 (40.6)	0.236	10,145 (29.6)	10,816 (31.5)	0.042
Malignant neoplasms	10,755 (31.1)	973,846 (47.5)	0.340	10,736 (31.3)	11,055 (32.2)	0.020
Systemic lupus erythematosus	1243 (3.6)	9889 (0.5)	0.222	1151 (3.4)	1393 (4.1)	0.037
Sjögren syndrome	944 (2.7)	15,415 (0.8)	0.152	907 (2.6)	1145 (3.3)	0.041
Chronic viral hepatitis C	600 (1.7)	11,598 (0.6)	0.110	596 (1.7)	661 (1.9)	0.014
Chronic viral hepatitis B	159 (0.5)	3308 (0.2)	0.054	155 (0.5)	171 (0.5)	0.007
HIV disease	158 (0.5)	6777 (0.3)	0.020	157 (0.5)	149 (0.4)	0.003
Systemic sclerosis	201 (0.6)	3043 (0.1)	0.072	192 (0.6)	223 (0.6)	0.012

*PSM*, Propensity score matching; *SMD*, standardized mean difference.

Primary outcomes were 6-month, 1-year, and 3-year incidences of myocardial infarction (MI), atrial fibrillation (AF), stroke, pulmonary embolism (PE), venous thromboembolism (VTE), and chronic kidney disease (CKD), excluding those with prior such diagnoses. Varicose vein diagnosis served as a negative control. Outcomes and comorbidities were identified utilizing validated ICD-10 codes, and the use of ICD-10-based algorithms has been validated for the identification of vasculitis in prior research.^3^^,^^4^ Hazard ratios were estimated using Cox proportional hazard and Kaplan-Meier survival analyses, with censoring. Statistical analyses were conducted within the TriNetX platform using Python version 3.7 (Python Software Foundation).

Further details on cohort creation and definitions are provided in Supplementary Supplement 1, available via Mendeley at https://data.mendeley.com/datasets/vc9sbk9cx5/1.

Among 33,824 individuals with SLV who were matched with controls, those with SLV had a higher risk of MI, AF, stroke, PE, VTE, and CKD across all time points (Table II). At 5 years from the index date, those with SLV had higher relative risks of MI (relative risk [RR]: 1.83; 95% CI: 1.65-2.03), AF (RR: 2.28; 95% CI: 1.97-2.64), stroke (RR: 2.21; 95% CI: 1.96-2.49), PE (RR: 1.44; 95% CI: 1.30-1.60), VTE (RR: 1.45; 95% CI: 1.33-1.57), and CKD (RR: 2.11; 95% CI: 1.97-2.27).

**Table II:** Occurrence and risk of systemic complications in patients with skin-limited vasculitis vs controls in a propensity score-matched analysis

Outcome	6-month risk				2-year risk				5-year risk			
	Cases, SLV cohort (*n* = 33,411)(%)	RD % (95% CI)	RR (95% CI)	*P* value	Cases, SLV cohort (*n* = 33,324) (%)	RD, % (95% CI)	RR (95% CI)	*P* value	Cases, SLV cohort (*n* = 33,824) (%)	RD, % (95% CI)	RR (95% CI)	*P* value
Myocardial infarction	255 (0.8%)	3.334 (2.588, 4.294)	3.484 (2.704, 4.490)	<.001	573 (1.8%)	1.972 (1.715, 2.267)	2.155 (1.873, 2.480)	<.001	919 (2.8%)	1.633 (1.473, 1.810)	1.829 (1.648, 2.031)	<.001
Atrial fibrillation	556 (1.7%)	3.915 (3.259, 4.705)	4.654 (3.366, 6.434)	<.001	831 (2.4%)	2.663 (2.340, 3.031)	2.688 (2.221, 3.253)	<.001	1035 (3.0%)	2.270 (2.035, 2.531)	2.279 (1.965, 2.643)	<.001
Stroke	294 (0.9%)	4.522 (3.470, 5.893)	4.728 (3.626, 6.165)	<.001	536 (1.7%)	2.498 (2.138, 2.920)	2.726 (2.330, 3.188)	<.001	778 (2.4%)	1.970 (1.749, 2.218)	2.206 (1.956, 2.487)	<.001
Pulmonary embolism	203 (0.6%)	2.156 (1.692, 2.747)	2.252 (1.767, 2.871)	<.001	484 (1.5%)	1.532 (1.331, 1.762)	1.678 (1.457, 1.932)	<.001	786 (2.4%)	1.286 (1.159, 1.428)	1.441 (1.297, 1.601)	<.001
Venous thromboembolism	346 (1.1%)	1.986 (1.661, 2.374)	2.073 (1.733, 2.480)	<.001	775 (2.5%)	1.442 (1.295, 1.606)	1.567 (1.406, 1.747)	<.001	1259 (4.1%)	1.305 (1.204, 1.416)	1.445 (1.330, 1.570)	<.001
Chronic kidney disease	737 (2.5%)	4.412 (3.732, 5.215)	4.599 (3.888, 5.440)	<.001	1465 (4.9%)	2.406 (2.192, 2.641)	2.625 (2.388, 2.885)	<.001	2183 (7.3%)	1.887 (1.761, 2.023)	2.113 (1.967, 2.269)	<.001
End-stage renal disease	147 (0.5%)	4.097 (2.846, 5.897)	4.246 (2.949, 6.113)	<.001	278 (0.8%)	1.896 (1.554, 2.315)	2.053 (1.681, 2.507)	<.001	392 (1.2%)	1.659 (1.413, 1.948)	1.835 (1.562, 2.157)	<.001
Varicose veins	34 (0.1%)	1.547 (0.905, 2.644)	1.626 (0.951, 2.779)	.073	126 (0.4%)	0.841 (0.664, 1.065)	0.947 (0.747, 1.200)	.650	214 (0.6%)	1.124 (0.925, 1.365)	1.232 (1.014, 1.497)	.239

*RD*, Risk difference; *RR*, relative risk; *SLV*, skin-limited vasculitis.

These findings align with prior evidence that localized immune complex deposition and cutaneous inflammation can trigger a systemic inflammatory cascade, leading to systemic complications.^5^ Further research is needed to clarify underlying relationships, as this study does not establish causality. Study strengths include its large sample size, covariate matching, and negative controls. Limitations include potential unmeasured confounders, observational experiment-related selection biases, inability to stratify results based on diagnostic characteristics, including histopathologic and immunofluorescence findings, and cohort and outcome misclassification risk from structured clinical data and ICD-10 codes. Further study is needed to identify best practices in monitoring and managing critical complications in skin-limited vasculitis, ensuring timely detection and treatment.

## Conflicts of interest

Dr Barbieri has received consulting fees from Honeydew Care and Sanofi Pasteur unrelated to the present submission. Drs Mahajan and Piette have no conflicts of interest to declare.

## References

[1] Watts R.A., Hatemi G., Burns J.C., Mohammad A.J. (2022). Global epidemiology of vasculitis. Nat Rev Rheumatol.

[2] Sunderkötter C.H., Zelger B., Chen K.R. (2018). Nomenclature of cutaneous vasculitis: dermatologic addendum to the 2012 revised international chapel hill consensus conference nomenclature of vasculitides. Arthritis Rheumatol.

[3] Kuang A., Xu C., Southern D.A., Sandhu N., Quan H. (2024). Validated administrative data based ICD-10 algorithms for chronic conditions: a systematic review. J Epidemiol Popul Health.

[4] Park J., Kwon S., Choi E.K. (2019). Validation of diagnostic codes of major clinical outcomes in a National Health Insurance database. Int J Arrhythmia.

[5] Mourino-Alvarez L., Perales-Sanchez I., Berna-Rico E. (2024). Association of the complement system with subclinical atherosclerosis in psoriasis: findings from an observational cohort study. J Invest Dermatol.

